# Serum Prealbumin Concentrations, COVID-19 Severity, and Mortality: A Systematic Review and Meta-Analysis

**DOI:** 10.3389/fmed.2021.638529

**Published:** 2021-01-26

**Authors:** Angelo Zinellu, Arduino A. Mangoni

**Affiliations:** ^1^Department of Biomedical Sciences, University of Sassari, Sassari, Italy; ^2^Discipline of Clinical Pharmacology, College of Medicine and Public Health, Flinders University and Flinders Medical Centre, Adelaide, SA, Australia

**Keywords:** prealbumin, COVID-19, disease severity, mortality, biomarker

## Abstract

Excessive inflammation and malnutrition are associated with coronavirus disease 2019 (COVID-19) severity and mortality. Combined biomarkers of malnutrition and inflammation, such as serum prealbumin, might be particularly attractive for early risk stratification. We conducted a systematic review and meta-analysis of studies reporting serum prealbumin in patients with COVID-19. We searched PubMed, Web of Science and Scopus, between January and November 2020, for studies reporting data on serum prealbumin, COVID-19 severity, defined as severe illness, prolonged viral load, receiving mechanical ventilation or admitted to intensive care unit (ICU), and mortality. Nineteen studies in 4,616 COVID-19 patients were included in the meta-analysis. Pooled results showed that serum prealbumin concentrations were significantly lower in patients with severe disease and non-survivors (standard mean difference, SMD, −0.92, 95% CI, −1.10 to −0.74, *P* < 0.001). Extreme heterogeneity was observed (*I*^2^ = 77.9%; *P* < 0.001). In sensitivity analysis, the effect size was not significantly affected when each study was in turn removed (range between −0.86 and −0.95). The Begg's (*P* = 0.06) and Egger's *t*-tests (*P* = 0.26) did not show publication bias. Pooled SMD values were significantly and negatively associated with age (*t* = −2.18, *P* = 0.045) and C-reactive protein (*t* = −3.85, *P* = 0.002). In our meta-analysis, lower serum prealbumin concentrations were significantly associated with COVID-19 severity and mortality. This combined marker of malnutrition and inflammation might assist with early risk stratification and management in this group.

## Introduction

Coronavirus disease 2019 (COVID-19), the condition responsible for the current global pandemic, is caused by the severe acute respiratory syndrome coronavirus 2 (SARS-CoV-2). COVID-19 severity and adverse clinical outcomes are positively associated with the presence of excessive systemic inflammation and immune response, and consequently with oxidative stress, coagulation disorders, and multiorgan failure (1–3). There is also increasing evidence that patients with more severe forms of COVID-19 are at risk of malnutrition, and that malnutrition itself is associated with adverse clinical outcomes (4). Pending the development of effective vaccines and in the absence of effective pharmacological treatments, with the exception of the glucocorticoid dexamethasone (5, 6), the identification of early markers of disease severity would assist with the appropriate selection of COVID-19 patients that benefit from intensive treatment and monitoring. This would also streamline specific care pathways, with positive effects on resource management and health care costs. Given the pathophysiological role of inflammation and nutrition in patients with COVID-19, markers that provide a combined assessment of these processes might be particularly useful in terms of predictive capacity and appropriate clinical decisions. The protein prealbumin, also known as transthyretin, is a negative acute phase-reactant produced in the liver that acts as a transport protein for thyroxine and is used as a marker of nutrition. Compared to albumin, prealbumin has a relatively short half-life, between 2 and 3 days vs. 20 days, is catabolized in the kidney and is not significantly affected by the presence of protein-losing enteropathy (7–9). Serum prealbumin concentrations <10 mg/dL have been shown to be associated with malnutrition, hospital length of stay, and mortality in other disease states (10–12). Given the capacity of low serum prealbumin concentrations to indicate the presence of a systemic inflammatory state and/or malnutrition, we conducted a systematic review and meta-analysis of the available evidence on the clinical implications of this protein specifically in patients with COVID-19.

## Methods

### Search Strategy, Eligibility Criteria, and Study Selection

We searched, using the terms “prealbumin” or “transthyretin” and “coronavirus disease 19” or “COVID-19,” the electronic databases PubMed, Web of Science, Scopus, and Google Scholar, between January and November 2020, to identify peer-reviewed studies reporting serum prealbumin concentrations, measures of COVID-19 severity, specifically the presence of clinically severe illness, prolonged viral load, need for mechanical ventilation or admission to intensive care unit (ICU), and mortality. The references of the articles identified were also searched for additional studies. Eligibility criteria were as follows: a) reporting continuous data regarding serum prealbumin concentrations in COVID-19, (b) investigating COVID-19 patients with different disease severity or clinical outcomes, particularly mortality, (c) investigating adult patients, (d) written in English, and (e) full-text available. Two investigators independently screened the abstracts. If relevant, they independently reviewed the full articles. The Newcastle-Ottawa scale was used to assess study quality by evaluating the cohort selection, cohort comparability on the basis of the design or analysis, how the exposure was determined and how the outcomes of interest were evaluated. Studies with a score of ≥6 indicated high quality (13).

### Statistical Analysis

Standardized mean differences (SMD) and 95% confidence intervals (CIs) were calculated to build forest plots of continuous data and to evaluate differences in serum prealbumin concentrations between COVID-19 patients with low vs. high disease severity or survivor vs. non-survivor status. A *P* < 0.05 was considered statistically significant. If concentrations were reported as median and interquartile range (IQR), the corresponding mean and standard deviation were calculated (14). Between-study heterogeneity in SMD values was assessed using the Q-statistic (*P* < 0.10 indicated significance). Inconsistency across studies was assessed using the *I*^2^ statistic (*I*^2^ < 25% indicated no heterogeneity; *I*^2^ between 25 and 50%, moderate heterogeneity; *I*^2^ between 50 and 75%, large heterogeneity; and *I*^2^ > 75%, extreme heterogeneity) (15, 16). A random-effects model was used, in the presence of high heterogeneity, to calculate the pooled SMD values and 95% confidence intervals. The influence of each study on the overall effect size estimate was investigated using sensitivity analysis, by sequentially excluding one study at a time (17). The associations between study size and magnitude of effect were analyzed using the Begg's adjusted rank correlation test and the Egger's regression asymmetry test, at the *P* < 0.05 level of significance, to assess the presence of potential publication bias (18, 19). The Duval and Tweedie “trim and fill” procedure was used to further test the effect of publication bias (20). This method recalculates a pooled SMD by incorporating the hypothetical missing studies as though they actually existed, to augment the observed data so that the funnel plot is more symmetric. Statistical analyses were performed using Stata 14 (STATA Corp., College Station, TX, USA). The study was fully compliant with the PRISMA statement on the reporting of systematic reviews and meta-analyses (21).

## Results

### Literature Search and Study Selection

**Figure 1:**
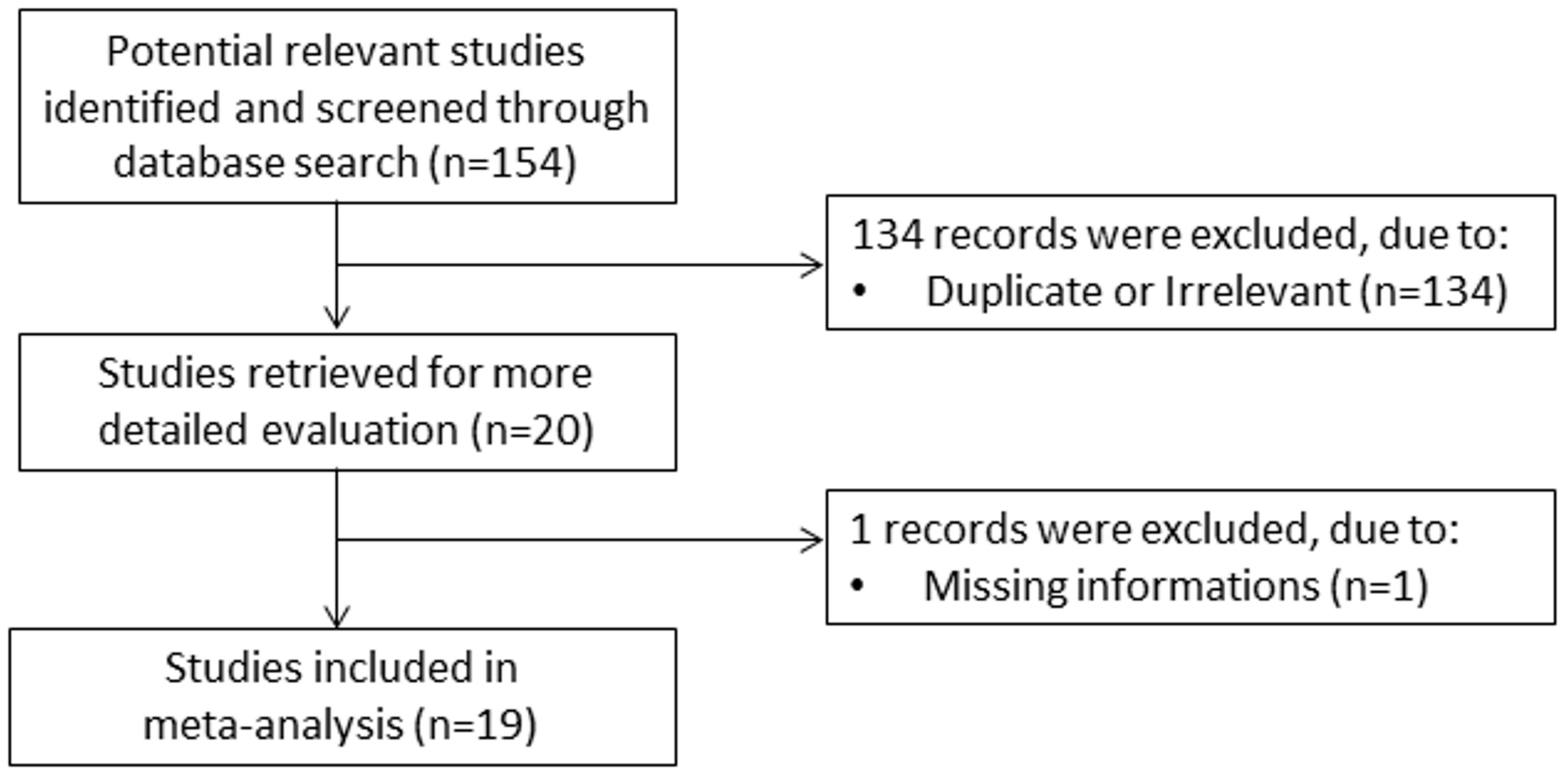
Flow chart of study selection.

A flow chart describing the study screening and selection is presented in Figure 1. From a total of 154 studies initially identified, 134 were excluded because they were either duplicates or not relevant. After a full-text review of the remaining 20 articles, one study was further excluded because of missing data, leaving 19 studies for analysis (22–40). The characteristics of these studies, all conducted in China, are described in Table 1. Overall, they included 4,616 COVID-19 patients, 3,502 (48% males, mean age 52 years) with low severity or who survived and 1,114 (61% males, mean age 64 years) with high severity or who died. Three studies were prospective (24, 30, 36),14 were retrospective (22, 23, 25–29, 31, 33, 34, 37–40), whereas the remaining two did not explicitly state the study design (32, 35). Disease severity, based on current clinical guidelines, was assessed in 11 studies (22, 23, 25, 28, 30, 33, 35, 37–40), prolonged viral clearance in two (26, 36), transfer to ICU in one (29), survival in three (27, 31, 32), presence of acute respiratory distress syndrome in one (34), and multiple end points in one (24).

**Table 1:** Characteristics of the selected studies in COVID-19 patients, according to disease severity and survival status.

				**Milder disease or survival**				**Severe disease or death**			
**References**	**Study design**	**Outcome**	**NOS (stars)**	***n***	**Age (Years)**	**Gender (M/F)**	**Prealbumin mg/dL (Mean ± SD)**	***n***	**Age (Years)**	**Gender (M/F)**	**Prealbumin mg/dL (Mean ± SD)**
Chen Z et al. (22)	R	Severe Non-severe	6	615	54	282/333	19.2 ± 10.3	221	63	129/92	10.9 ± 6.5
Duan J et al. (23)	R	Severe Non-severe	6	328	44	170/158	22.7 ± 7.7	20	58	14/6	14.8 ± 5.4
Feng X et al. (24)	P	Good outcome Pooroutcome^*^	7	94	63	58/36	12.0 ± 4.5	20	69	13/7	11.9 ± 7.3
Fu HY et al. (25)	R	Severe Non-severe	3	33	40	NR	23.9 ± 7.4	4	66	NR	15.2 ± 6.9
Gao C et al. (26)	R	Prolonged load Non-prolongedload^**^	3	63	59	26/37	13.7 ± 8.7	49	68	25/24	10.1 ± 4.7
Guo J et al. (27)	R	Survivor Non-survivor	6	43	60	22/21	17.8 ± 9.8	31	68	21/10	9.2 ± 5.0
Ji M et al. (28)	R	Severe Non-severe	6	70	NR	NR	15.3 ± 6.9	51	NR	NR	12.1 ± 55
Li G et al. (29)	R	ICU Non-ICU	6	312	49	131/181	18.3 ± 7.3	211	62	119/92	14.7 ± 6.2
Li L et al. (30)	P	Severe Non-severe	6	60	51	NR	18.3 ± 5.4	12	45	NR	10.8 ± 3.2
Li T et al. (31)	R	Survivor Non-survivor	7	66	NR	NR	21.3 ± 5.2	9	NR	NR	11.4 ± 6.0
Luo Y et al. (32)	NR	Survivor Non-survivor	6	986	59	476/510	21.9 ± 7.7	129	70	87/42	13.7 ± 4.9
Sun L et al. (33)	R	Severe Non-severe	7	40	40	23/17	21.0 ± 3.9	15	67	8/7	13.0 ± 6.4
Wu C et al. (34)	R	ARDS NoARDS	7	117	48	68/49	13.5 ± 5.2	84	59	60/24	10.2 ± 3.8
Xue G et al. (35)	NR	Severe Non-severe	4	56	61	30/26	17.5 ± 9.2	58	64	34/24	9.8 ± 6.2
Xue J et al. (36)	P	Prolonged load Non-prolongedload^**^	6	35	42	23/12	19.7 ± 6.5	13	61	6/7	16.4 ± 8.3
Yang P et al. (37)	R	Severe Non-severe	4	65	41	32/33	21.8 ± 5.7	68	60	40/28	7.0 ± 10.4
Zhang XY et al. (38)	R	Severe Non-severe	6	89	66	35/54	13.8 ± 5.5	21	71	17/4	8.3 ± 3.6
Zhang Y et al. (39)	R	Severe Non-severe	6	84	44	29/55	20.4 ± 8.2	31	65	20/11	12.2 ± 7.4
Zhao X et al. (40)	R	Severe Non-severe	6	346	59	175/171	15.6 ± 8.1	67	65	37/30	10.4 ± 5.4

*ARDS, acute respiratory distress syndrome; ICU, intensive care unit; Non-severe, patients with mild or moderate disease; NOS, Newcastle-Ottawa quality assessment scale; NR, not reported; P, prospective; R, retrospective; Severe: patients with severe or criticaldisease*.

* *Patients that were discharged, those with non-severe condition, and those not requiring mechanical ventilation were considered to have a good outcome. Patients requiring mechanical ventilation and those who died were considered to have a pooroutcome*.

** *Viral clearance*.

### Meta-Analysis

**Figure 2:**
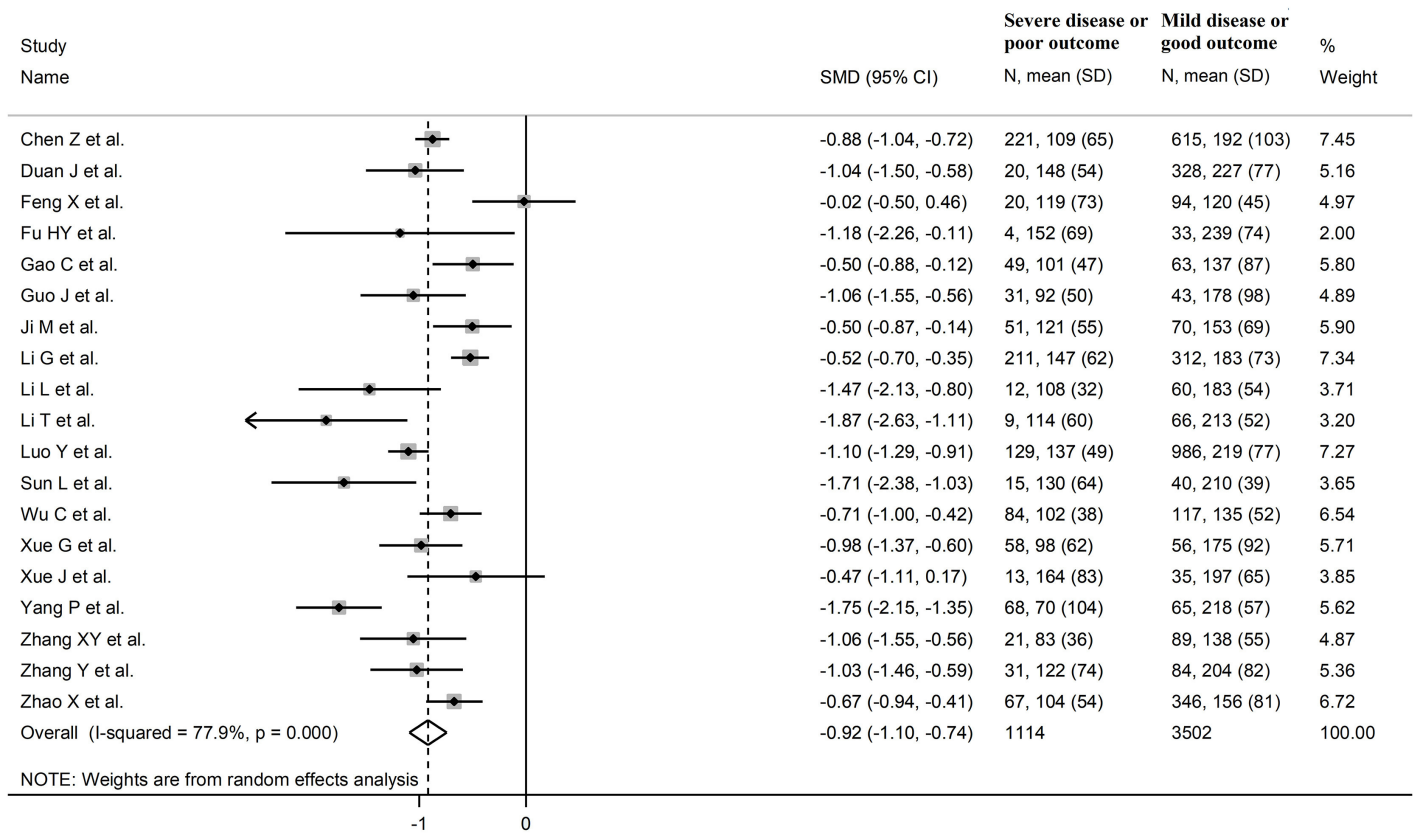
Forest plot of studies examining serum prealbumin concentrations in patients with COVID-19.

**Figure 3:**
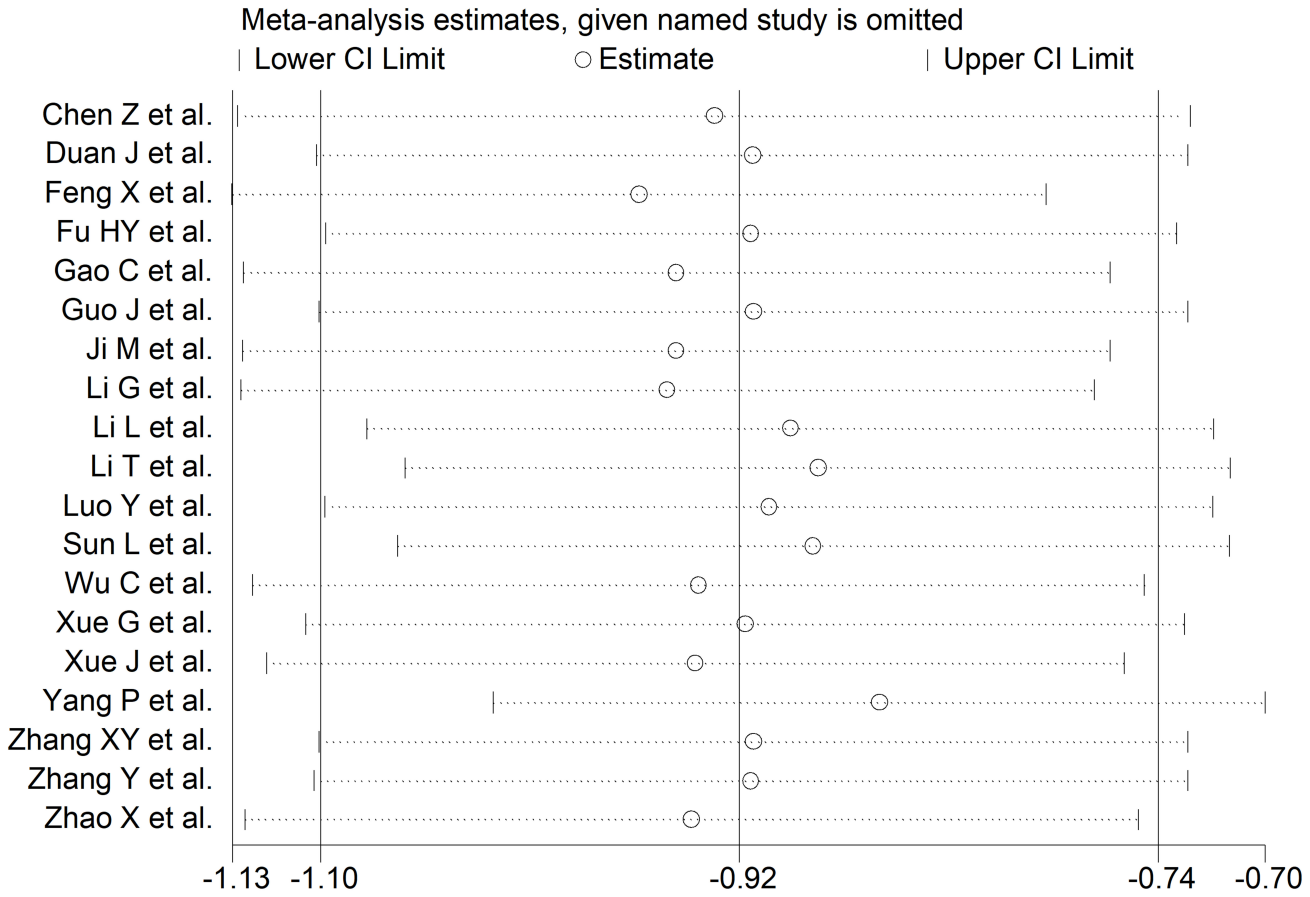
Sensitivity analysis of the association between serum prealbumin and COVID-19. The influence of individual studies on the overall standardized mean difference (SMD) is shown. The middle vertical axis indicates the overall SMD and the two vertical axes indicate the 95% confidence intervals (CI). Hollow circles represent the pooled SMD when the remaining study is omitted from the meta-analysis. The two ends of each broken line represent the 95% CIs.

**Figure 4:**
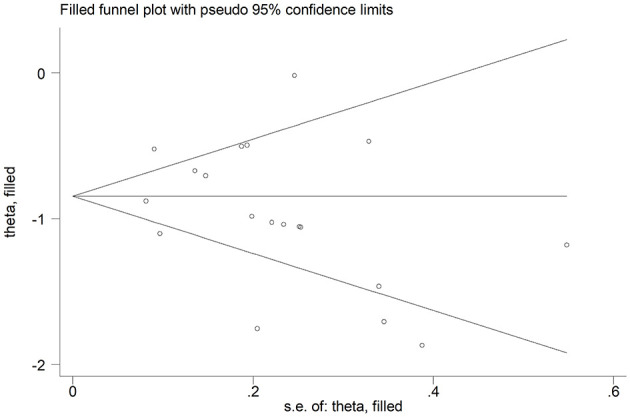
Funnel plot of studies investigating low vs. high severity or survivor vs. non-survivor status after trimming and filling. Dummy studies and genuine studies are represented by enclosed circles and free circles, respectively.

The overall SMD values in patients with mild vs. severe disease or survivor vs. non-survivor status in the 19 studies are described in Figure 2. In all studies, patients with severe disease or non-survivor status showed lower serum prealbumin concentrations when compared to those with milder disease or survivor status (mean difference range, −1.87 to −0.02). However, in two of these studies the difference was not statistically significant (24, 36). The pooled results showed that serum prealbumin concentrations were significantly lower in COVID-19 patients with severe disease or non-survivor status (SMD −0.92, 95% CI −1.10 to −0.74, *P* < 0.001) (Figure 2). There was extreme heterogeneity between studies (*I*^2^ = 77.9%; *P* < 0.001). In sensitivity analysis, the effect size was not affected when each study was in turn removed (effect size range, between −0.86 and −0.95) (Figure 3). The Begg's (*P* = 0.06) and Egger's *t*-tests (*P* = 0.26) showed no evidence of publication bias. The trim-and-fill method did not identify any study that was missing or should be added (Figure 4).

**Figure 5:**
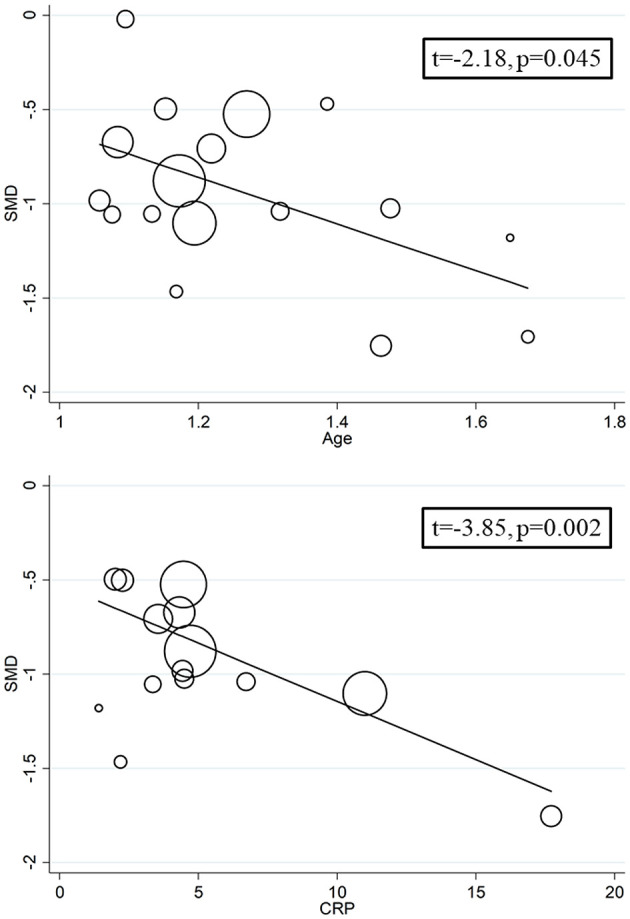
Univariate meta-regression analysis between effect size, age and C-reactive protein.

**Figure 6:**
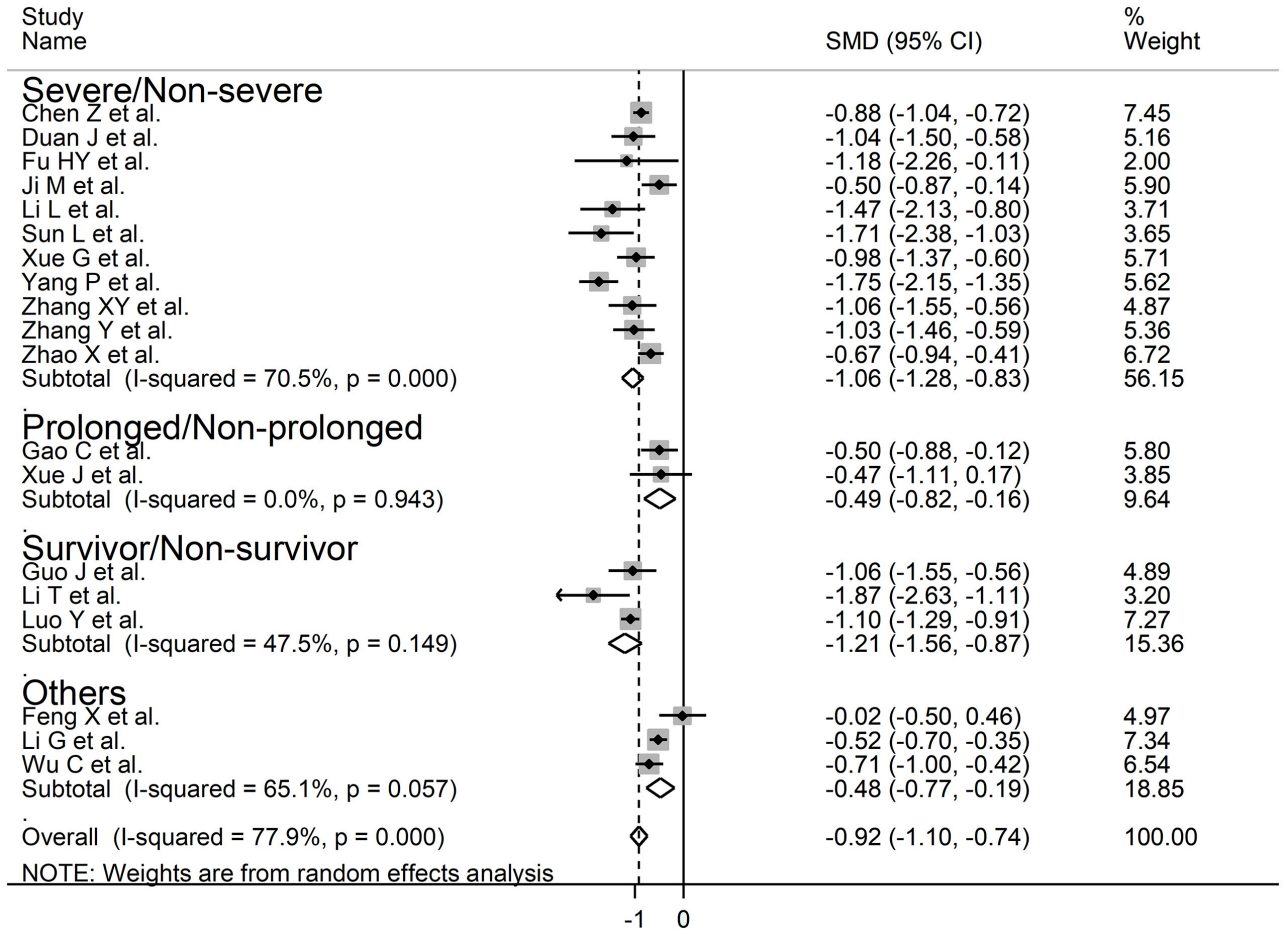
Forest plot of studies examining serum prealbumin concentrations and COVID-19 according to disease severity or outcome.

**Figure 7:**
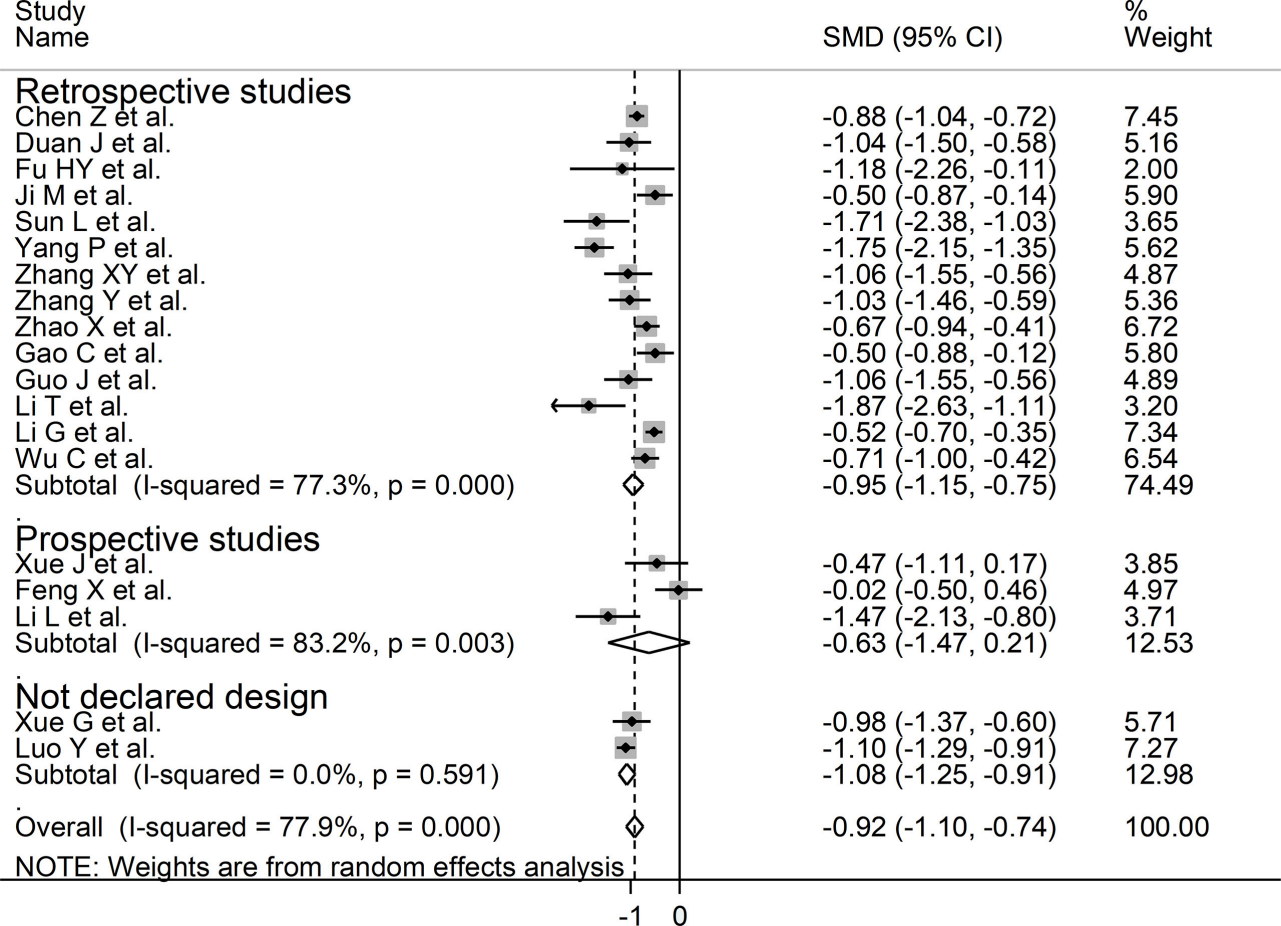
Forest plot of studies examining serum prealbumin concentrations in patients with COVID-19 according to study design (prospective vs. retrospective).

We investigated possible factors contributing to the observed between-study variance, particularly the effect of age, gender, specific end points, study design (retrospective vs. prospective), and the inflammation biomarker C-reactive protein, on SMD by univariate meta-regression analysis. Both age (*t* = −2.18, *P* = 0.045) and CRP (*t* = −3.85, *P* = 0.002) were significantly and negatively associated with the pooled SMD (Figure 5). By contrast, there were no significant correlations between SMD and gender (*t* = −0.83, *P* = 0.42). The pooled SMD value in studies assessing survival (−1.21, 95% CI −0.87 to −1.56, *P* < 0.001; *I*^2^ = 47.5%, *P* = 0.15) was lower than that observed in studies assessing disease severity (−1.06, 95% CI −0.83 to −1.28, *P* < 0.001; *I*^2^ = 70.5%, *P* < 0.001) and viral clearance (−0.49, 95% CI −0.16 to −0.82, *P* = 0.003; *I*^2^ = 0.0%, *P* = 0.94) however the difference was not statistically significant in meta-regression analysis (*t* = 1.69, *P* = 0.11, Figure 6). A relatively low heterogeneity was observed in studies assessing survival, *I*^2^ = 47.5%, and viral clearance, *I*^2^ = 0.0%, compared to that in studies assessing severity, *I*^2^ = 70.5%. No significant differences (*t* = 0.21, *P* = 0.84) were observed between pooled SMD values in retrospective (−0.95, 95% CI −0.75 to −1.15, *P* < 0.001; *I*^2^ = 77.3%, *P* < 0.001) and prospective studies (−0.63, 95% CI 0.21 to −1.47, *P* = 0.14; *I*^2^ = 83.2%, *P* = 0.003, Figure 7).

## Discussion

Our systematic review and meta-analysis showed that serum prealbumin concentrations were significantly lower in COVID-19 patients with severe disease, i.e., with clinically severe illness, prolonged viral load, receiving mechanical ventilation or admitted to ICU, or those that succumbed to the disease when compared to patients with milder forms of the disease or who survived. The observed SMD value, −0.92, suggests a large, biologically and clinically relevant, effect size (41). Despite the extreme between-study heterogeneity in sensitivity analysis the overall effect size was not significantly affected when individual studies were removed. Furthermore, there was no evidence of publication bias. Notably, the SMD was significantly and negatively associated with age and CRP concentrations but not with gender, type of end point studied (disease severity, viral load clearance, need for mechanical ventilation, admission to ICU, or survival), or study design (retrospective vs. prospective).

A reduction in serum prealbumin concentrations typically indicates the presence of acute inflammation and/or malnutrition, unlike other biomarkers such as CRP and procalcitonin which predominantly reflect the inflammatory burden (42–44). Its relatively short half-life makes it a suitable marker to assess and monitor rapid changes inflammatory burden and nutritional state. Normal serum prealbumin concentrations range between 16 and 35 mg/dL. Concentrations <10 mg/dL have been associated with malnutrition and adverse outcomes in non-COVID-19 patient groups (10–12). In particular, studies have reported that serum prealbumin can predict adverse outcomes in patients with burn injuries, respiratory disease, cardiac surgery, and systemic sclerosis (45–48). The results of our systematic review and meta-analysis expand the potential clinical applications of serum prealbumin as the early assessment of this parameter might assist with management decisions in hospitalized patients with COVID-19. This is particularly important given that SARS-CoV-2 is a relatively new virus and, consequently, the models of care for COVID-19 undergo regular review and update following the emergence of novel disease biomarkers and/or therapeutic strategies. Low serum albumin concentrations on admission might help to identify, together with other clinical and demographic characteristics, those patients that are more at risk of severe disease and/or transfer to ICU. Furthermore, unlike CRP and procalcitonin, they could guide nutritional intervention strategies as an important element of care (49).

The exact mechanisms responsible for the lower serum prealbumin concentrations observed in high-risk COVID-19 patients are unclear however they are likely related to the excess inflammation and cytokine release commonly observed in this group (50, 51). Prealbumin is a well-known negative acute-phase reactant, therefore its serum concentrations typically decrease during acute inflammatory processes (52). The significant negative associations observed between the SMD values and CRP concentrations in univariate meta-regression analysis support this hypothesis. There is also emerging evidence that malnutrition is a negative prognostic factor in COVID-19. For example, in a study of 348 hospitalized COVID-19 patients 139 (40%) had moderate-severe malnutrition. The latter group was characterized by older age, higher male prevalence, and higher CRP concentrations and had an increased risk of acute cardiac injury and mortality when compared to patients with mild malnutrition. In multivariate regression analysis, the controlling nutritional status score independently predicted mortality (odds ratio 1.41, 95% CI 1.09–1.82, *P* = 0.009) (53).

While the extreme between-study heterogeneity represents a potential limitation the overall effect size was not significantly affected in sensitivity analysis. Furthermore, no evidence of publication bias was observed. Notably, unlike age and CRP concentrations, the SMD values of serum prealbumin concentrations were not significantly associated with specific study end points (disease severity, viral load clearance, need for mechanical ventilation, admission to ICU, and survival) or design (prospective vs. retrospective). However, the relatively low heterogeneity observed in studies assessing survival and viral clearance suggests that the selection of specific end points may, at least partially, contribute to the observed between-study variance. It is also possible that other, unreported, factors might have contributed to the observed heterogeneity. Another potential limitation is that all selected studies were conducted in China. Therefore, additional studies in other ethnic groups and geographical locations are required to support the generalizability of the results. In conclusion, our systematic review and meta-analysis has shown that lower serum prealbumin concentrations are significantly associated with high disease severity and mortality in patients with SARS-CoV-2 infection. The measurement of serum prealbumin, singly or in combination with other clinical and demographic parameters, might represent a relatively inexpensive and easy to derive biomarker to guide clinical decisions in hospitalized patients with COVID-19.

## Data Availability Statement

The original contributions presented in the study are included in the article/supplementary material, further inquiries can be directed to the corresponding author/s.

## Author Contributions

AZ: initial idea and data collection and analysis. AZ and AM: data interpretation and writing—review & editing. AM: writing—first draft. All authors contributed to the article and approved the submitted version.

## Conflict of Interest

The authors declare that the research was conducted in the absence of any commercial or financial relationships that could be construed as a potential conflict of interest.
